# Exploring disparities in malnutrition among under-five children in Nigeria and potential solutions: a scoping review

**DOI:** 10.3389/fnut.2023.1279130

**Published:** 2024-01-05

**Authors:** Collins John, Bee Koon Poh, Muhammad Yazid Jalaludin, Godpower Michael, Idris Adedeji, Elizabeth Eberechi Oyenusi, Blessing Akor, Nkwoala C. Charles, Vanitha Buthmanaban, Leilani Muhardi

**Affiliations:** ^1^Department of Paediatrics, College of Health Sciences, University of Jos, Jos, Nigeria; ^2^Nutritional Sciences Programme and Centre for Community Health Studies (ReaCH), Faculty of Health Sciences, Universiti Kebangsaan Malaysia, Kuala Lumpur, Malaysia; ^3^Department of Paediatrics, University Malaya Medical Centre, Kuala Lumpur, Malaysia; ^4^Department of Family Medicine, Aminu Kano Teaching Hospital, Kano, Nigeria; ^5^Department of Paediatrics, Abubakar Tafawa Balewa University Teaching Hospital, Bauchi, Nigeria; ^6^Department of Paediatrics, College of Medicine, University of Lagos/Lagos University Teaching Hospital, Lagos, Nigeria; ^7^Department of Family Medicine, University of Abuja, Abuja, Nigeria; ^8^Department of Community Medicine, University of Abuja, Abuja, Nigeria; ^9^Department of Human Nutrition and Dietetics, Michael Okpara University of Agriculture, Umudike, Nigeria; ^10^FrieslandCampina, Amersfoort, Netherlands; ^11^FrieslandCampina, AMEA, Singapore

**Keywords:** children below 5 years, Nigeria, poor quality of complementary feedings, multiple undernutrition issues, targeted interventions

## Abstract

**Introduction:**

Triple burden of malnutrition in children remains a significant public health issue. This scoping review aims to assess the information on undernutrition, micronutrient deficiencies and the quality of complementary feeding in various regions in Nigeria.

**Methods:**

A literature search was conducted using PubMed and Google Scholar databases from January 1, 2018 to January 31, 2023 to include studies focusing on 0 to 5 years old children in Nigeria, reporting data on nutritional status, nutrient deficiencies, and published in English.

**Results:**

73 out of 1,545 articles were included. Stunting remained alarmingly high ranging from 7.2% (Osun, South West) to 61% (Kaduna, North Central), while wasting varied from 1% (Ibadan, South West) to 29% (FCT Abuja, Central) and underweight from 5.9% (Osun, South West) to 42.6% (Kano, North West) respectively. The overall prevalence of anemia and vitamin A deficiency ranged between 55.2 to 75.1 % and 5.3 to 67.6%, respectively. Low rates of achieving minimum dietary diversity and minimum meal frequency were reported across different states depicting the suboptimal quality of complementary feeding. The prevalence of overweight/obesity ranged from 1.5% (Rivers, South South) to 25.9% (Benue, North Central).

**Conclusion:**

Multiple early childhood malnutrition issues exist with a wide disparity across states in Nigeria, particularly in the Northern region. Targeted nutrition interventions must be implemented to improve the situation.

## Introduction

Malnutrition is defined as a “*pathological state resulting from inadequate or excess nutrition*” (1). According to the World Health Organization (WHO), ‘poor nutrition status’ is the sole important threat to the world’s health, with adequate nutrition being the critical element in helping individuals to live healthy and productive lives. Malnutrition impacts the intergenerational economic growth of a country. Morbidity related to malnutrition leads to a loss in human capital through an education gap and a resultant low-skilled workforce owing to poor cognitive development and reduced school attainment (2). Inadequate food intake and poor dietary quality are directly or indirectly responsible for causing poor health, with the top 6 of the 11 global risk factors associated with dietary imbalances (3).

Malnutrition is expressed through undernutrition (stunting, wasting, and underweight) and/or overnutrition, which is related to a high intake of protein and energy (1). The WHO in 2021 has defined complementary feeding indicators, including minimum dietary diversity (MDD), minimum meal frequency (MMF), and minimum acceptable diet (MAD) (4), as essential in the early years of life to the formation of eating habits that may eventually have short- and long-term implications on the child’s health (5). Severe acute malnutrition (SAM) or protein energy malnutrition (PEM) is a life-threatening condition requiring urgent attention. Over the years, it has been known by different names such as protein-calorie malnutrition, PEM, oedematous malnutrition, nutritional oedema, severe wasting, or based on clinical manifestations including marasmus, kwashiorkor, or marasmic kwashiorkor (6).

Globally there are over 150 million stunted children, 50 million wasted children, and 38 million underweight children (6). In addition, over 40 million children under the age of 5 years are overweight (6). While more countries are dealing with several forms of malnutrition indices, individual children are known to suffer from more than one form of malnutrition indicators across the world (7).

Nigeria is one of the top 10 countries with malnutrition in children aged under 5 years (7). It has the second-highest burden of stunted children in the world and a higher-than-the-world average child-wasting prevalence (8). Within Nigeria, nearly half of all under-5 children were stunted in the North East and North West geopolitical zones, while the rest of Nigeria contributed to 22.0 % of under-5 stunting prevalence (9).

Despite the availability of studies on stunting, there is a paucity of information on other nutritional issues across all the regions of Nigeria. Hence, the objective of this study was to review the current evidence on nutritional status, nutrient intake, and quality of complementary feeding in under-5 children across all the geopolitical zones and states of Nigeria.

## Methodology

### Search strategy

**Figure 1:**
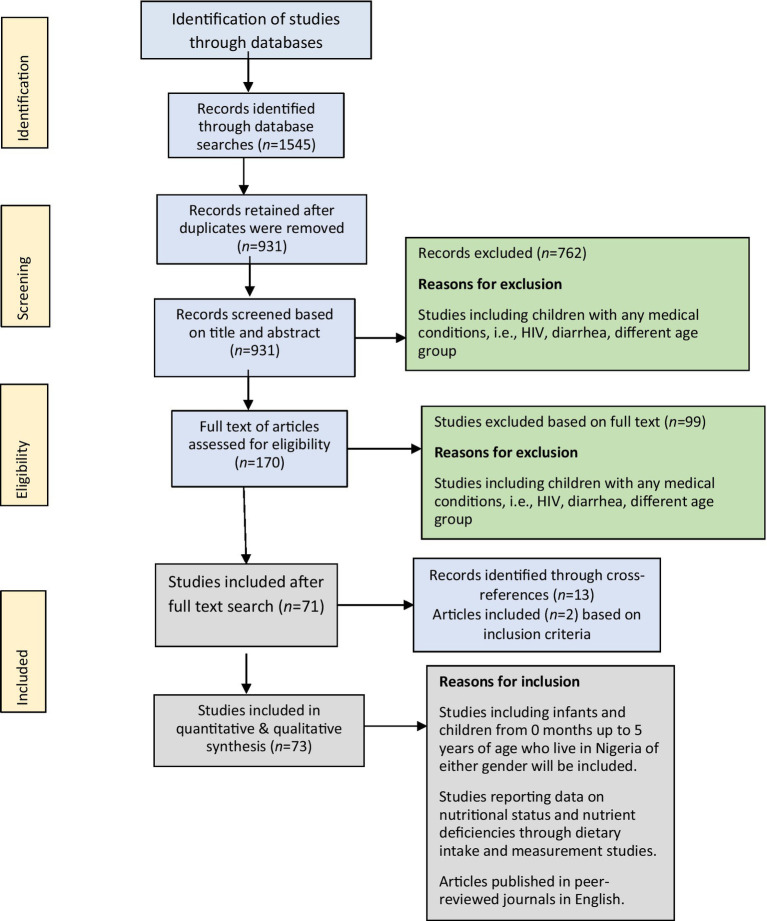
PRISMA flow diagram of study search and selection process.

Literature searches were carried out using the PubMed and Google Scholar databases. The main search terms used in the literature search were malnutrition, nutritional deficiency, Nigeria, stunting, wasting, or underweight. The details of the search are provided in Table 1. The results are reported according to the PRISMA guidelines for scoping reviews (Figure 1).

**Table 1:** Summary of search strategy and results.

Search engine	Search key string	Number of results returned	Articles screened
Google Scholar	Nigeria* AND (child* OR toddler OR infant) AND (inadequate OR problem OR intake OR condition OR situation OR status) AND nutrition* AND (“under-five” OR “pre-school”)	5,370	470
	Nigeria* AND (child* OR toddler OR infant) AND (inadequate OR problem OR intake OR condition OR situation OR status) AND nutrition* AND (“under-five” OR “pre-school”) -”		299
	Nigeria* AND (child* OR toddler OR infant OR nursery OR baby) AND (inadequate OR condition OR status OR deficien* OR insufficient OR maln* OR undern* OR wast* OR stunt*) AND nutritional* AND (“pre-school” OR “under-five”) -primary		343
PubMed	((Nigeria*[Title/Abstract]) AND (child*)) AND (nutrition)		235
	Nigeria* AND (child* OR toddler OR infant) AND (malnutrition OR nutritional deficiency) AND nutrition* AND (“under-five” OR “pre-school”)		198

### Screening of articles and selection of studies

For all the articles, the search results were extracted and imported into reference management software (Mendeley). All duplicates were removed, and a researcher screened the results based on manuscript’s title and abstract based on the following criteria:

#### Inclusion criteria

- Studies including infants and children from 0 to 60 months of age of either gender living in Nigeria.
- Studies reporting data on nutritional status and nutrient deficiencies through dietary intake and measurement studies.
- Baseline data from intervention studies.
- Specific case reports if relevant.
- Review articles
- Articles published from 01-01-2018 to 31-01-2023 to focus on recent available data.
- Articles published in peer-reviewed journals in the English language.

#### Exclusion criteria

- Studies including children with any medical conditions, such as sickle-cell anemia, human immunodeficiency virus (HIV), and diarrhea.
- Grey literature such as reports.
- Single case reports on a specific disease.

Two authors (CJ and LM) reviewed the retrieved full-text articles for final inclusion in the study. If there were any disagreements in the selection of the articles, a third author (PBK or MYJ) acted as a referee to decide whether the article under consideration should be excluded or included. LM extracted date from the eligible retrieved articles to provide extraction of information. No assessment on data quality was implemented.

### Data extraction

Data for the following outcomes of interest were extracted from relevant research articles: participant characteristics (age, sex, geographical location, socioeconomic status, weight, and height); nutritional status (stunting, wasting, underweight,) acute malnutrition, overweight, and obesity; nutrient deficiencies biochemical analysis (including iron deficiency anemia, vitamin A, and zinc deficiency); from dietary intake studies including energy; macronutrients (protein, total fat, total calorie); and micronutrients (iron, zinc, vitamin A, calcium, magnesium, phosphorus, vitamin D, B-vitamins, vitamin C, zinc, folate).

Results were pooled by region to facilitate regional comparison. Available data describing the estimation of malnutrition, nutrient status, and dietary quality, including differences by age, gender, and setting (urban vs. rural), are also discussed in the narrative findings for each outcome. The data in the review is incorporated as a range from the lowest to the highest percentage values or as per geopolitical zones in Nigeria, namely North Central, North East, North West, Central, South East, South South, South West or the year of study or publication.

### Definitions of important terms

In this study, WHO definitions for various malnutrition status were used. Stunting in a child is defined with a height-for-age Z-score (HAZ) of below minus two standard deviations (−2SD) from the median; wasting as a weight-for-age Z-score of below minus two standard deviations (−2SD) from the median. An underweight is weight-for-height Z-score is less than minus two standard deviations (−2SD) from the median and could be a composite extraction of both stunting and wasting child’s status; a child with severe stunting has HAZ < −3SD and moderately stunting as −3SD ≤ HAZ ≤ −2SD. The same classification also holds for other anthropometric indicators of undernutrition (1). The WHO in 2021 has defined complementary feeding indicators, including MDD (percentage of children 6–23 months of age who consumed foods and beverages from at least 5 out of 8 defined food groups during the previous day), MMF (percentage of children 6–23 months of age who consumed solid, semi-solid or soft foods the minimum number of times or more during the previous day), and MAD (percentage of children 6–23 months of age who consumed at least MDD and MMF during the previous day) (4). Inadequate energy intake used in the study is defined as energy intake less than 80% of recommended daily intake (570–1742 kcal). Similarly, inadequate intake of other micronutrients is also defined as less than 80% of recommended daily nutrient intake (10). Severe and moderate anaemia is defined as <7 g/dl and 7.0–9.9 g/dl, respectively (11).

## Results

Seventy-three articles were included in the study based on the inclusion–exclusion criteria. Along with the national prevalence, data was also available from 20 states covering all six geopolitical zones of Nigeria. Amongst the included articles, a majority of studies (84.9%) reported data for the prevalence of undernutrition status (stunting, wasting, and underweight), overweight, and obesity. Articles reporting stunting prevalence (61.6%) were the highest in number, while that reporting wasting (53.4%) and underweight (46.6%) prevalence was relatively less. Only six articles provided information on SAM/PEM (8.2%). Approximately 28.8% of articles reported nutrient intake data, 47.6% reported anemia prevalence, 33.3% reported vitamin A deficiency prevalence, 19.0% reported zinc deficiency, 14.3% reported iron and vitamin D deficiency, while only 1 article each was identified reporting the prevalence of vitamin B12, vitamin C, and potassium deficiency.

On average, only 15% of the articles mentioned the year the study was conducted. Some of the selected articles with year of publication after January 1, 2018, did however cite data from earlier date including those from 2003.

### Severe acute malnutrition/protein-energy malnutrition

The average national prevalence of SAM/PEM in Nigeria was 8.8% (12, 13); however, the prevalence data reported a wide region-wise variance in prevalence, ranging from 1.9 to 46.0% (14–17) (Supplementary Table 1A). The highest SAM/PEM prevalence was reported from the South West and North West geopolitical zone of Nigeria (15, 16). In the Osun state (South West), the prevalence of Marasmus, Kwashiorkor, and Marasmus-Kwashiorkor were as low as 2.0, 3.0, and 2.0%, respectively (15).

### Overall prevalence of undernutrition (stunting, wasting, and underweight)

**Figure 2:**
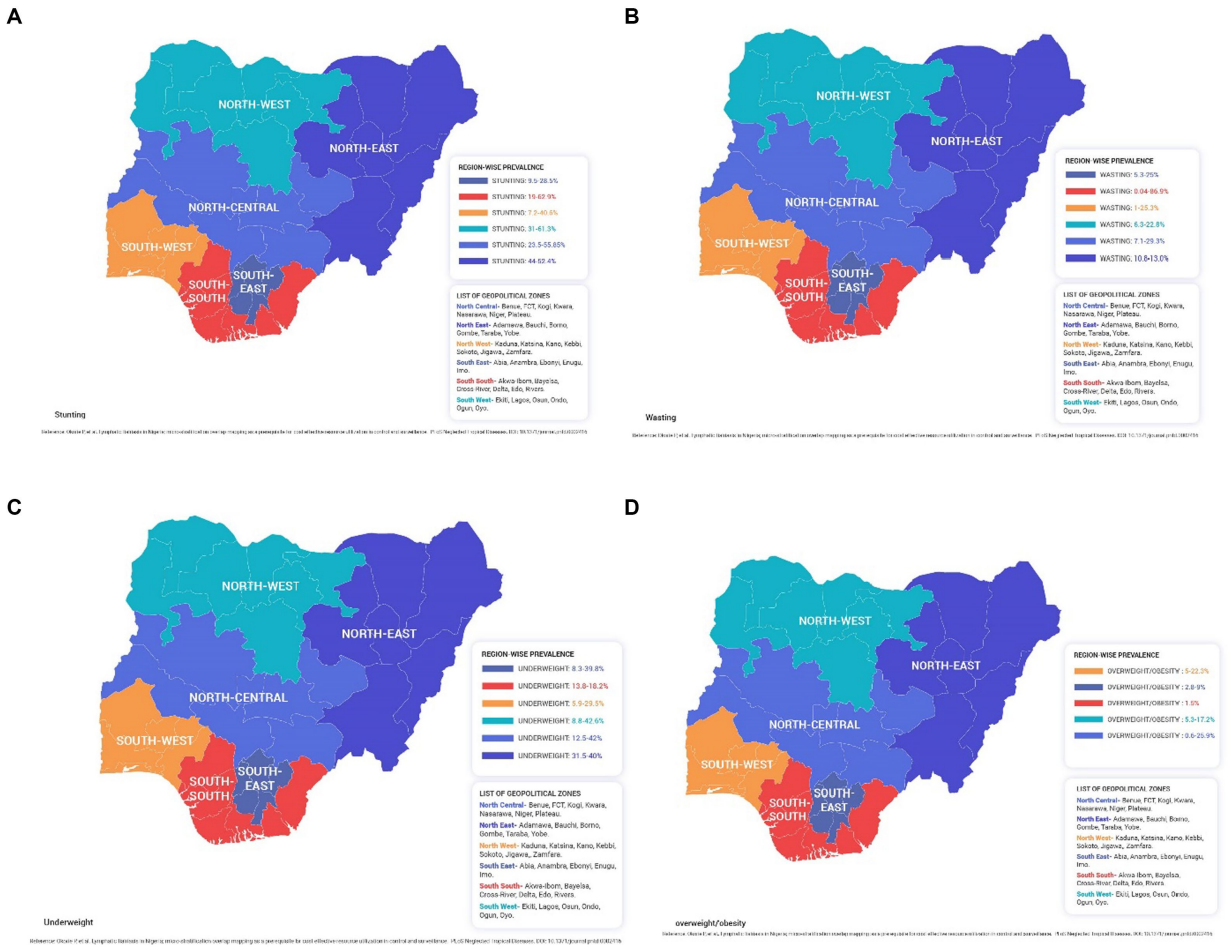
Malnutrition map of Nigeria for **(A)** stunting, **(B)** wasting, **(C)** underweight, **(D)** overweight and obesity.

From several retrieved literature, the national prevalence of undernutrition has been reported to decline from 2003 to 2013 and 2018 (45% vs. 37% vs. 36.8 % for stunting, 13% vs. 8% vs. 6.7% for wasting, and 27.2% vs. 28.9 % vs. 21.4% for underweight) (18–20). The regional prevalence of stunting, wasting, and underweight reported a difference from the national prevalence in Nigeria, as seen in the geopolitical zone mapping of Nigeria (Figure 2).

As per National Demographic Health Survey (NDHS) 2018, the national stunting prevalence among under-5 children in Nigeria was reported to be 36.1 % (21). The prevalence by zone reported stunting to be the highest in Kaduna in North West (61.3%) (22) and Kwara in the North Central zone (55.8%; Supplementary Table 2) (23). The lowest prevalence of stunting was reported from Osun state in the South West region (7.2%) (24). The severity of stunting varied among states from 3.4% in Rivers (South West) (25) to 54.5% in Kaduna (North West) (26). The prevalence of moderate stunting ranged from 8.2% in Anambra (South East) (27) to 28.8% in Kaduna (North West) (26) (Supplementary Table 2). From 2003 to 2018, the national prevalence of severe stunting reduced from 22.8 to 16.9% (28).

Several states in Nigeria reported a high prevalence of wasting, which was above the national prevalence in Nigeria, including Anambra (South East; 30%) (29), FCT, Abuja (North Central; 29.3%) (30), Bida, Niger (North Central; 27.8%) (31), Ondo (South West; 25.3%) (32), Enugu (South East; 25.0%) (33), Osun (South West; 24.0%) (34), and Kano (North West; 22.8%) (35). The lowest prevalence of wasting (1.0%) was seen in Ifedore, Ondo state (South West) (36). However, none of these studies cited the year of data collection.

The underweight prevalence in Nigerian states ranged from 5.9% in the Osun state (South West) (34) to 42.6% in the North West zone of Nigeria (37). Several states reported underweight prevalence above the national prevalence, with the highest prevalence in North West including Kano State (around 41.9%-42.6%) (35, 37), followed by Enugu state (South East; 39.8%) (24).

There are more than 10 reported risk factors for undernutrition, among others: malaria and anemia (38), having more than four children and religious belief (39), having a respiratory or diarrheal infection (28, 40). However, immunization status, maternal education, parental income, maternal height and body mass index (BMI); as well as having antenatal care less than four times are among commonly reported risk factors (28, 39–43).

### Urban vs. rural prevalence of undernutrition

**Figure 3:**
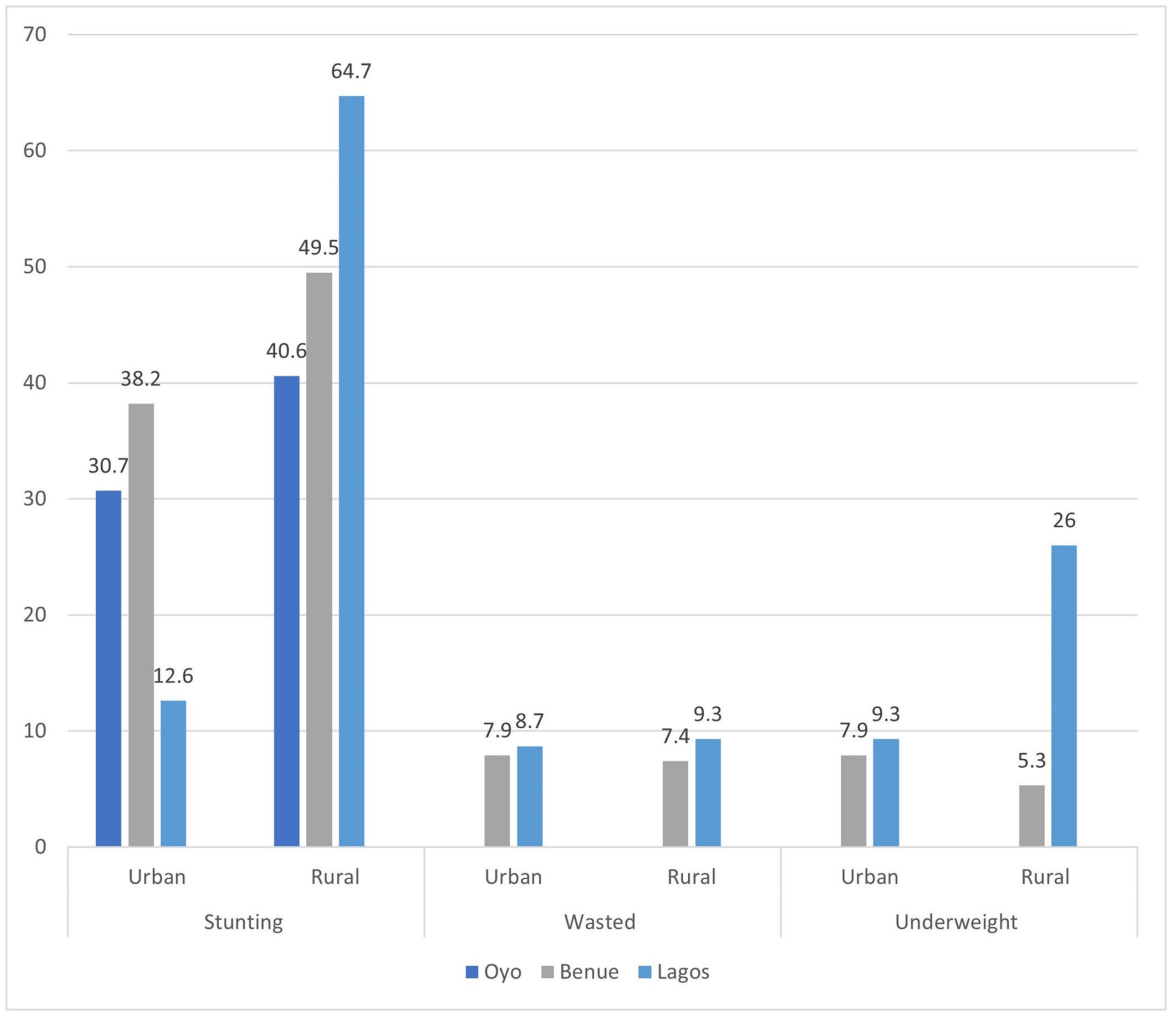
Urban–rural prevalence of undernutrition across a few states in Nigeria.

It was seen that the overall national prevalence of stunting, wasting, and underweight in urban areas of Nigeria ranged between 26.8–30.6%, 10.5–35.6%, and 23.0–31.2%, respectively, while in the rural regions, it was 44.8–72.8%, 10.5–69.9%, and 35.5–72.9%, respectively (Supplementary Table 3). The highest prevalence of stunting was reported in the urban Ibadan, Oyo state (44) and rural regions of Lagos (39). State-wide prevalence of wasting was almost similar in rural regions compared to urban regions, with the highest prevalence reported from Lagos state (urban: 8.7%; rural: 9.3%) (38). The Ibadan urban region in Oyo state showed a wasting prevalence of 1.8% which was much below the national prevalence (10). The prevalence of underweight was considerably higher in rural compared to urban (19, 37, 45–47) (Figure 3).

### Gender-based prevalence of undernutrition

There was no definitive pattern has been noticed in the prevalence of gender-based undernutrition (Supplementary Table 4). A few studies have reported higher stunting prevalence in males compared to females. The regional prevalence differs from the national prevalence in that the males depict a higher prevalence of stunting and wasting but not underweight (48–51). In contrast, wasting prevalence was slightly higher amongst females than males (22, 50, 52, 53). There were no differences between males and females regarding underweight prevalence (18, 45) (Supplementary Table 4).

In males, wasting prevalence ranged from 2.5 to 50.0%; underweight ranged from 6.0 to 78.0%, while in females, wasting prevalence was reported from 8.0 to 61.0% and underweight from 7.0 to 61.4% (16, 22, 48, 50, 54, 55).

### Age-based prevalence of undernutrition

Between 2008 and 2018, the NDHS data reported that the prevalence of stunting among children 6–11 months ranged from 28.9 to 35.4%, while the prevalence of stunting among 0–23 months old was 39.1%, and among 24 to 59 months was 53.3% (55). The prevalence of underweight was stable of around 10% among infants 0–6 months and 6–11 months. However, this number doubled to around 20.0% in older children of 12–60 months.

**Figure 4:**
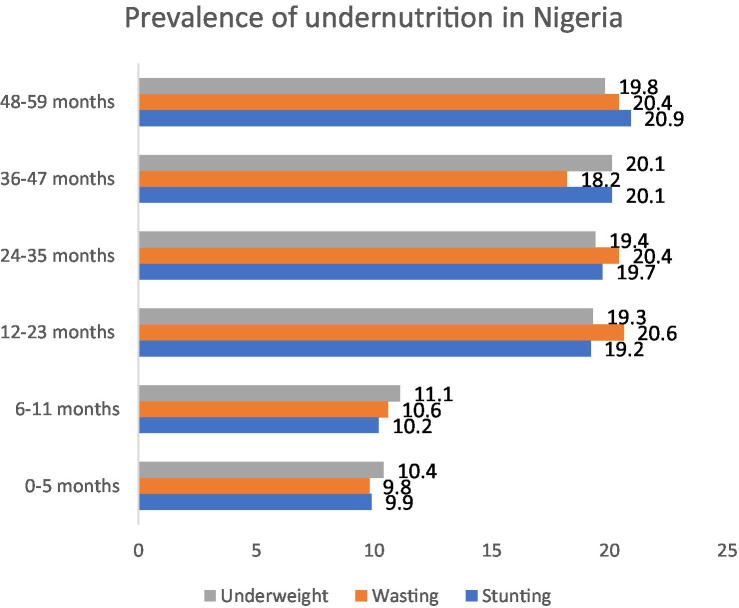
Prevalence of undernutrition among children according to age.

The prevalence of wasting was the highest among children 12–35 months (Figure 4).

### Overweight and obesity

The data collected across 13 states reported the national prevalence of obesity in 2013 to be 4.0% (20, 43), while in 2018, it declined to 2.0%. The overweight prevalence ranged from 1.5 to 25.2%, while obesity ranged from 0.6 to 25.9% among under-5 children in different regions of Nigeria (21, 25, 30, 56, 57). The prevalence of overweight in male children in Ondo and Benue states was 9.8 and 10.5%, while in female children, it was 10.7 and 8.4%, respectively (53, 58) (Supplementary Table 5).

### Dietary intake

The prevalence of inadequate energy intake ranged from 18.0 to 34.0% (36, 59). A study in Ibadan, Oyo State (South West), reported an inadequate intake of fat (92.0%), protein (46.0%), and carbohydrates (30.0%) (10, 11). Other studies reported that the prevalence of inadequate intake for different micronutrients in under-5 children including zinc 32.0–91.0%, potassium 91.0%, vitamin A 82.0%, vitamin C 80.0%, iron 74.0%, and folate 44.0% (59, 60).

### Anemia and iron deficiency

Data from the NDHS shows the national prevalence of anemia among children under-5 years of age in Nigeria has slightly declined from 67.0% reported in 2013 to 58% in 2018 (20). However, the anemia prevalence has shown an upward trend in urban regions from 55.2% in 2010 to 62.0% in 2018, while in rural regions; the prevalence remains similar which was reported around 75.1% in 2010 and 72.5% in 2018 (61).

**Figure 5:**
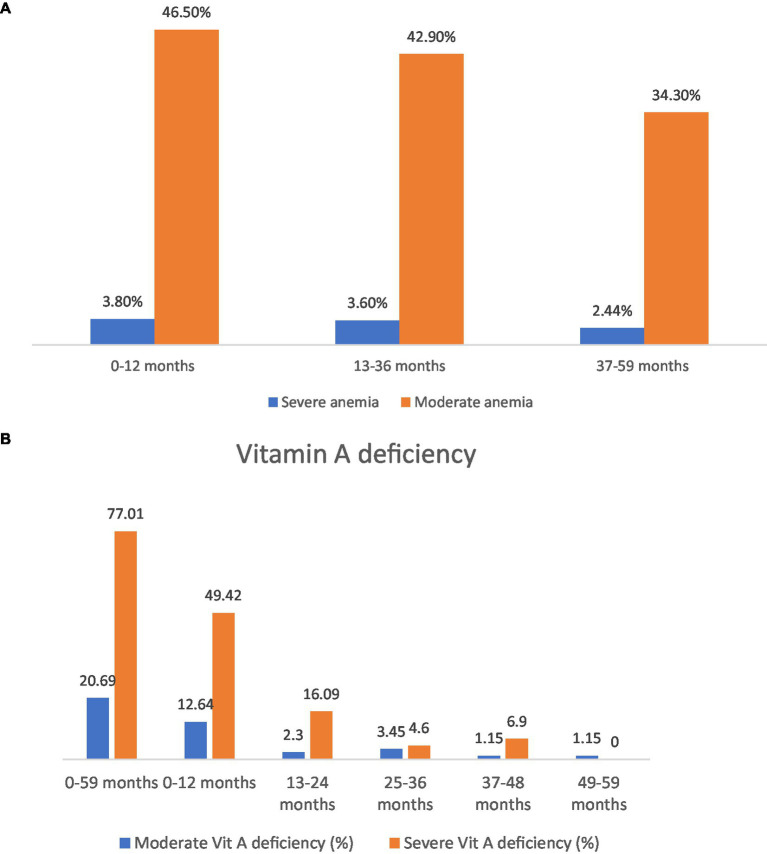
**(A)** Anemia prevalence in different age groups. **(B)** Severe vs. moderate vitamin A deficiency in different age groups.

The prevalence of severe and moderate anemia in males was reported to be 3.6 and 43.3%, respectively, and in females, the prevalence of severe and moderate anemia was 2.9 and 38.4%, respectively (11). The highest prevalence of severe and moderate anemia was reported in children under 1 year (11) (Figure 5A). Moderate iron deficiency ranged from 1.1 to 32.2%, while severe deficiency ranged from 1.1 to 67.8% (16). Having vitamin A deficiency and malaria are some of the risk factors reported to be associated with anemia (62, 63).

### Other micronutrient deficiencies

Vitamin A deficiency ranged from 5.3 to 67.6% (24, 64), with a severe deficiency of 77.0 and 20.7% of moderate deficiency (Figure 5B) (16). The prevalence of vitamin A deficiency in a study of children 3 to 5 years of age ranged from 8.0 to 10.0% (65). Factors reported to be associated with vitamin A deficiency are anaemia (16) and low consumption of vitamin A-rich foods (26).

Based on baseline data of an intervention study among toddlers in Lagos (South West), it was reported a deficiency of vitamin D (9.0–37.0%), folate (8.0–21.1%), and vitamin B12 (7.1%) (66). Moderate vitamin D deficiency ranged from 1.15 to 24.14%, while severe deficiency ranging from 2.3 to 50.6% was reported in Kaduna state (16). Low serum zinc was reported in 12.4–26.0%, being higher in males (32.1%) than in females (19.1%) (33, 67).

### Quality of complementary feed (MDD, MAF, and MAD)

The proportion of children achieving MDD ranged from 3.0 to 55.2% in Nigeria (37, 54, 68–71) (Table 2). Dietary diversity was classified into low (meals comprising 1–6 food groups) and high (meals comprising 6–12 food groups). The prevalence of low and high dietary diversity reported in one study was 68.5 and 31.5%, respectively (22). While another study reported a total of 85.5% among 6- to 24-month-old children fulfilled the MDD, with the highest prevalence seen in 9- to 11-month-old infants (36.0%) (70).

**Table 2:** Proportion of children who meet the minimum dietary diversity, minimum meal frequency, and minimum acceptable diet across different regions in Nigeria.

Region	State	Year of data collection	Minimum dietary diversity (%)	Minimum meal frequency (%)	Minimum acceptable diet (%)	Reference
North East		2016-2017	31.7	47.0	15.8	(36)
North West		2016-2017	37.5	40.1	14.4	(36)
	Kano	2019	31.3			(63)
	Kaduna	2019	28.0			(63)
North Central		2016-2017	41.5	46.5	16.6	(36)
South East		2016-2017				
		2016-2017	52.3	48.1	16.9	(36)
South West		2016-2017	46.8	33.4	12.8	(36)
	Ogun	2019	85.5	66.4	90.8	(65)
	Ijero, Ekiti	NA			40.7	(66)
	Ikole, Ekiti	NA			29.4	(66)
South South	Edo	NA		76.3		(51)
		2016-2017	55.2	43.1	18.9	(36)

NA: not available.

The proportion of children achieving MMF ranged from 33.4 to 76.3% (37, 54, 68–70). In a study by Samuel and Ibidapo, the highest prevalence of MMF was 30.0% in infants 9 to 11 months of age. A high prevalence of MAD (90.8%) was reported in children in Osun state (70). Several factors reported in this review to be associated with poor quality of complementary foods are antenatal care visit, child’s welfare clinic visit, mother’s work place and education, as well as household size (70).

## Discussion

This scoping review summarizes available evidence on the burden of malnutrition in under-5 children in various regions of Nigeria as well as the nutrient deficiency and quality of complementary feeding. It highlights the disparities across regions, age groups and gender across several indicators of malnutrition, such as stunting, wasting, underweight, and anemia. While a total of 73 studies were identified, most reported data on nutritional status; only 6 reported the prevalence of SAM/PEM, and a few studies (less than 30%) revealed information on nutrient intake/deficiency or quality of complementary feeding.

The prevalence of stunting, wasting, and underweight in under-5 children was high, exhibiting considerable variations across different zones. The North West and North Central zones have been reported to have the highest prevalence of SAM, stunting, wasting, underweight, iron deficiency anemia, and moderate/severe vitamin D deficiencies and the lowest proportion of children under 6 months who met the MDD and MAD. In contrast, the prevalence of undernutrition was found to be generally lower in South East Nigeria compared to other zones (72).

The high prevalence of poor nutritional status among under-5 children in the Northern zones could be attributed to the militant insurgency, banditry, and cattle rustling hindering a large number of farmers and livestock herders from accessing their farmlands for almost a decade. These disastrous scenarios not only lead to restrictions on agricultural produce but also severe food insecurity, affecting many households residing in these zones, particularly in the rural regions (9, 22, 37, 48, 55). This situation also drives more consumption of staple foods as accessible sources of energy (37). In addition, there were some food taboos for children and pregnant women that restrict their access to certain foods, such as eggs which were more prominent in the Northern region (37). It was also clearly seen that undernutrition prevalence was more pronounced in rural regions than in urban regions.

More than half of the under-5 children have anemia with a higher prevalence reported in rural areas than in urban regions. Studies have shown that anemia in children leads to poor cognition, school achievement, and more behavioral issues as they grow, particularly into middle childhood (73). In several cases, it may lead to irreversible damage to the child’s development (73); hence, efforts to alleviate the anemia burden are necessary in Nigeria. This review also noted that a substantial prevalence of vitamin A deficiency in Nigeria.

Undernutrition particularly in the first 2 years of life, has been reported to lead to an impairment of their ability to resist disease (74, 75), undertake physical activity, and progress in study and school (74, 75). The impact of not achieving growth potential impedes the cognitive development of the child, with enormous and adverse implications on well-being and economic prospects as the child grows into adulthood. Undernutrition in the early years of life leads to reduced educational completion, lower economic productivity, increased morbidity, and reduced life expectancy (9). Studies have shown that children who suffer from undernutrition are not able to complete their education and show deficiencies in vocabulary and mathematics, along with other learning or intellectual disabilities, which leads to reduced economic productivity in adulthood (73, 78). This depletion of human capital not only poses social and economic challenges in disadvantaged communities of Nigeria but also hinders the overall economic and social progress of the country in general.

In addition, this report also shows the high prevalence of overweight and obesity in several states in North Central and South West Nigeria. This confirms the presence of a triple burden of malnutrition among children under 5 years of age in Nigeria which further worsens the dire situation of these children as resources need to be allocated to address these three problems simultaneously (79, 80).

The review reported a high prevalence of underweight and wasting among children between 12-35 months while the highest prevalence of stunting was in children 24-59 months old. In addition, the highest prevalence of severe and moderate anemia was reported in children under 1 year of age. These three conditions could be the root cause of why the prevalence of stunting remained and will continue to be high in Nigeria as has been shown in other countries (81). Studies have shown that underweight/wasting in the early life can persist into failure to thrive and will continue to stunting at a later age (82, 83).

Undernutrition among under-5 children is driven by a mesh of influencing factors, including cultural, behavioral, and environmental factors. The quality of complementary feeding practices is one of the important factors in determining the child’s nutritional status. The 6- to 24-month age group is characterized by increased energy expenditure and need for nutritional requirements through complementary feeding. Inappropriate complementary feeding practices significantly contribute to undernutrition among children contributing as a major cause of disease burden, especially in developing countries (84). Maternal characteristics such as education and BMI were frequently reported in this review as influencing factors for stunting and the quality of complementary foods. This points out the importance to have better education and nutrition status of the mothers to ensure a healthy period during the first 1,000 days. In addition, malaria and anemia seem to counter-influence each other, thus it is important to address both factors together whenever one of these health issues occurred.

### Potential solutions to overcome the disparities in nutritional issues

Possible interventions that have been shown to reduce the prevalence of malnutrition should be able to address the problem at different levels. These are itemized as follows: (1) at an individual level: increased uptake and attendance of antenatal care visits, lower average number of birth and mandatory vaccination for children and women (81, 85), and improvement of nutrition and health knowledge among girls and pre-pregnant women (81); (2) at the household level: change in behavior to include the use of clean fuel for cooking, encouragement for more hygienic behavior including a reduction in the practice of open defecation (81); (3) at regional level: food subsidies or food-based intervention in the northern region of Nigeria has been shown to simultaneously overcoming multiple nutritional challenges in the area; and (4) at the national level to support at least four times minimal antenatal care, address anemia and malaria (86), to enhance awareness of vaccination in young children (85), create more equitable household economic growth (81, 87), and build infrastructure for greater access to improve toilet facilities (81, 88).

It is crucial to prioritize efforts that enhance, among others, access to nutritious food, improve healthcare services, and implement effective, targeted interventions. Furthermore, there is a need for more monitoring efforts to evaluate the effectiveness of these interventions as a basis for further refinement of the interventions in the future.

## Strengths and limitations

This scoping review offers a comprehensive assessment of the nutritional status of children in Nigeria, as well as various related indicators, providing invaluable insights to guide future research endeavours. Furthermore, it highlights the need to invest in public policies and socioeconomic strategies that can foster improved nutrition status and address the underlying issues. This study meticulously collates an array of essential undernutrition parameters, complementary feeding indicators, and dietary intake status pertaining to under-5 children in Nigeria. Drawing from recent data sourced from national health surveys, regional surveys, and other pertinent studies, a spectrum of prevalence rates spanning from low to high has been thoughtfully incorporated based on geopolitical zone. To enhance accessibility, the amassed data has been meticulously organized and presented in a user-friendly mapping format.

Due to the heterogeneity of studies, the absence of precise study years, and limited results for some regions, the undertaking of a comprehensive meta-analysis or applying statistical analysis to individual study results was not feasible.

### Areas for further research

There is a dearth of data available for micronutrient intake and deficiency. For example, in this review, there were no data retrieved on nutrients related to immunity, such as vitamin C and E (89), or nutrients that are important for bone growth, such as calcium, phosphorus, magnesium (90), and also nutrients that are commonly reported to be deficient in other populations (such as pregnant women in Nigeria) like thiamine and riboflavin (91, 92). For macronutrients, total protein and LCPUFA intake in under-5 children, for example, was notably absent in all of the selected studies.

## Conclusion

The culmination of this comprehensive analysis has brought to the forefront a multitude of pressing nutritional problems, prominently underscored by the pervasive triple burden of malnutrition encompassing stunting, overweight, and anemia. The northern zones in Nigeria have emerged as particularly vulnerable, grappling with a more acute undernutrition crisis as evidenced by the array of indicators examined.

Gender differentials further paint a nuanced picture, whereby stunting exhibited a higher prevalence among males, while the prevalence of wasting in several states, was skewed toward females. The widespread occurrence of anemia across diverse geographical regions, reveals noteworthy disparities between urban and rural areas. The high prevalence of vitamin A and D deficiency, along with inadequate nutrient intake, were also notable.

Compounding these challenges, a significant proportion of children were found to lack access to a diverse range of food groups, and fell short of consuming the recommended number of meals per day in their complementary diets.

In light of the far-reaching impact of malnutrition, encompassing the loss of human capital and reduced economic productivity across Nigeria, the imperative for targeted and expeditious nutrition interventions becomes evident. Swift and concerted action are crucial to rectify the prevailing disparities in nutrition status among young children in Nigeria, securing a healthier and more prosperous future for the nation at large.

## Data availability statement

The original contributions presented in the study are included in the article/Supplementary material, further inquiries can be directed to the corresponding author.

## Author contributions

CJ: Conceptualization, Data curation, Investigation, Methodology, Writing – review & editing, Formal analysis. BP: Conceptualization, Data curation, Investigation, Writing – review & editing, Formal analysis. MJ: Conceptualization, Formal analysis, Writing – review & editing, Methodology. GM: Formal analysis, Methodology, Writing – review & editing, Data curation. IA: Formal analysis, Methodology, Writing – review & editing, Investigation. OE: Formal analysis, Investigation, Writing – review & editing, Data curation. BA: Formal analysis, Writing – review & editing, Methodology. NC: Formal analysis, Methodology, Writing – review & editing, Data curation. VB: Writing – review & editing. LM: Writing – review & editing, Conceptualization, Data curation, Formal analysis, Investigation, Methodology, Supervision.
