# Supplementary material for: Exploring disparities in malnutrition among under-five children in Nigeria and potential solutions: a scoping review
Source: Front Nutr. 2024 Jan 5;10:1279130. doi: 10.3389/fnut.2023.1279130 (PMC10796494; doi:10.3389/fnut.2023.1279130)
Supplement: Supplementary file 1 [file Data_Sheet_1.pdf]

Supplementary Table 1a.: Prevalence of severe acute (SAM) and protein energy malnutrition (PEM) by states across Nigeria

| **Region** | **State** | **Year of data collection** | **SAM/PEM** | **Reference** |
|---|---|---|---|---|
| North West | Kaduna | NA | 36.0 | Tanimu et al, 2021 (16) |
| North Central | FCT-Abuja | NA | 2.9 | Oyinwola et al., 2022 (14) |
| South East | Enugu | NA | 1.9 | Ekechukwu et al, 2022 (17) |
| South West | Osun | 2017 | 46.0 | KAB O, 2022 (15) |

Supplementary Table 1b.: Prevalence of Stunting, Wasting, and Underweight by states across Nigeria

| **Region** | **State** | **Year of data collection** | **Stunting (%)** | **Wasting (%)** | **Underweight (%)** | **Reference** |
|---|---|---|---|---|---|---|
| North East | - | 2016-2017 | 52.4 | 13.0 | 40.0 | Moeteke et al, 2019 (37) |
| | Sokoto | 2017 | 31.0 | - | 24.0 | Manyong et al, 2021(58) |
| | Kebbi | 2017 | 33.9 | - | 22.8 | Manyong et al,2021(58) |
| | Kaduna | 2019 | 42.9 | - | 22.2 | Resnick et al, 2021 (68) |
| | Kano | 2016-2017 | 58.5 | 12.9 | 42.6 | Moeteke et al 2019 (37) |
| | | 2017 | 48.6 | 22.8 | 41.9 | Maje et al, 2019 (35) |
| | | 2019 | 46.0 | - | 26.9 | Resnick et al, 2021 (68) |
| | Kaduna and Jigawa | NA | 61.3 | 6.3 | 8.8 | Kehinde et al 2020 (22) |
| | Kaduna (North & South) | NA | 58.6 | - | - | Tanimu et al 2021 (16) |
| North Central | Benue | 2017 | 31.7 | - | 12.5 | Manyong et al 2021(58) |
| | | NA | 44.4 | - | - | Seer-Uke et al, 2021(56) |
| | Bida, Niger | NA | 23.5 | 27.8 | 27.4 | GO et al, 2022 (31) |
| | | 2016-2017 | 34.9 | 7.1 | 19.6 | Moeteke et al 2019 (37) |
| | Kwara | 2014 | 55.8 | - | - | Fadare et al, 2019 (23) |
| | FCT, Abuja | NA | 41.0 | 29.3 | 42.0 | Idowu et al, 2020 (30) |

| | | | | | | |
|---|---|---|---|---|---|---|
| South East | - | 2016-2017 | 16.9 | 8.2 | 13.7 | Moeteke et al, 2019 (37) |
| | Ebonyi | 2017 | 28.5 | | 12.5 | Manyong et al, 2021 (58) |
| | Enugu | 2018 | 9.5 | 5.3 | 8.3 | Ajah et al,2021 (41) |
| | | NA | 23.4 | 6.7 | 15.6 | Amadi et al, 2018 (49) |
| | | NA | - | - | 39.8 | Ekechukwu et al, 2022 (17) |
| | | NA | - | 25 | 28.8 | Ezeofor et al, 2020 (52) |
| South West | - | 2016-2017 | 19.4 | 8.9 | 16.6 | Moeteke et al 2019 (37) |
| | Ondo | 2017 | 18.6 | 25.3 | 29.5 | Oludu et al 2019 (32) |
| | Ifedore, Ondo | NA | 23.0 | 1.0 | 20.0 | Adewole et al 2022 (36) |
| | Ondo | 2019 | 16.7 | 13.8 | 21.7 | Akande et al 2019 (53) |
| | Ibadan, Oyo | NA | 27.4 | 1.8 | - | Yahaya et al (10) |
| | Ogun | 2017 | 28.6 | - | 16.4 | Okike et al 2021,(93) |
| | | 2017 | 32.5 | - | 17.7 | Manyong et al 2021(58) |
| | Ibadan (North), Oyo | NA | 30.7 | - | - | Adepoju et al, 2018 (44) |
| | Ido, Oyo | NA | 40.6 | - | - | Adepoju et al, 2018 (44) |
| | Osun | NA | 12.9 | 24.0 | 5.9 | Abolurin et al, 2018 (24) |
| | | NA | 7.2 | 9.2 | 8.4 | Abolurin et al, 2020 (24) |
| | | 2016 | 28 | - | - | Afolami et al,2021(65) |
| | Lagos | NA | 28 | 8.3 | 14.3 | Senbanjo et al, 2019 (39) |
| South South | - | 2016-2017 | 19 | 6.8 | 13.8 | Moeteke et al, 2019 (37) |
| | Akwa Ibom | NA | 37.4 | 13.1 | 18.2 | Alamu et al, 2020 (33) |
| | Edo | 2018 | - | 0.1 | - | Ajakaye et al 2020 (63) |

Supplementary Table 2: Prevalence of Severe and Moderate Undernutrition by States across Nigeria

| Region | State | Year of study | Stunting (%) | | Wasting (%) | | Underweight (%) | | Reference |
|---|---|---|---|---|---|---|---|---|---|
| | | | **Severe** | **Moderate** | **Severe** | **Moderate** | **Severe** | **Moderate** | |
| **North West** | Kaduna | | 54.5 | 28.8 | 66.7 | 13.6 | | | Abdullahi et al, 2021 (26) |
| | Kaduna | NA | 44.8 | 13.8 | 31.4 | 16.3 | 36.1 | 25.3 | Tanimu et al, 2021(16) |
| | Kebbi | 2017 | 28.1 | 15.8 | | | 8.4 | 14.4 | Manyong et al, 2021 (58) |
| | Sokoto | 2017 | 15 | 15.8 | | | 6.3 | 17.7 | Manyong et al 2021 (58) |
| **North Central** | Benue | 2017 | 18.6 | 13.1 | | | 4.1 | 8.4 | Manyong et al, 2021 (58) |
| | Bida, Niger State | NA | 5.7 | 17.8 | 6.5 | 21.3 | 10.4 | 17.0 | GO et al, 2022 (31) |
| | FCT | NA | 12.9 | | 5.7 | | 8.8 | | Idowu et al, 2020 (30) |
| | FCT Abuja | NA | | 44.7 | 37.2 | 29.2 | 46.0 | 37.8 | Idowu et al, 2020 [37] |
| **South East** | Ebonyi | 2017 | 13.9 | 14.6 | | | 4.6 | 8.9 | Manyong et al, 2021(58) |
| | Anambra state | NA | | 8.2 | | 8.2 | | | Onuekwe et al ,2018 (29) |
| **South South** | Rivers | NA | 39.1 | 8.7 | 27.6 | 8 | 13.5 | 24.2 | Nkeiruka et al, 2018 (33,47) |
| | Rivers | NA | 3.4 | 10.2 | 1.5 | 7.3 | 3.4 | 7.1 | Okari et al, 2019(25) |
| | Akwa Ibom | NA | 17.6 | 19.8 | 6.9 | 6.2 | 6.9 | 11.3 | Alamu et al, 2020 (33) |
| **South West** | Ogun | 2017 | 13.6 | | | | 16.4 | | Okike et al, 2021(93) |
| | Ogun | 2017 | 13.6 | 15.0 | | | 5.3 | 11.1 | Manyong et al, 2021 (58) |
| | Ibadan, Oyo state | NA | 11.9 | 15.5 | 2.4 | 6.4 | | 3.5 | Yahaya et al, 2021 (10) |

Supplementary Table 3: Prevalence of Stunting, Wasting, and Underweight in Urban and Rural areas in Nigeria

| **State** | **Year of data collection** | **Stunting (%)** | | **Wasting (%)** | | **Underweight (%)** | | **Reference** |
|---|---|---|---|---|---|---|---|---|
| | | **Urban** | **Rural** | **Urban** | **Rural** | **Urban** | **Rural** | |
| **NATIONAL PREVALENCE** | | | | | | | | |
| **Nigeria** | 2013 | 27.2 | 72.8 | 35.6 | 64.4 | 31.2 | 68.8 | Agu et al, 2022 (46) |
| | 2016-2017 | 30.6 | 49.3 | 10.5 | 10.9 | 23.0 | 35.3 | Moeteke et al, 2019 (37) |
| | 2018 | 28.1 | 71.9 | 30.1 | 69.9 | 27.1 | 72.9 | Tesfaw et al, 2021 (19) |
| | 2002-2020 | 26.8 | 44.8 | - | - | - | - | Ekholuenetale et al, 2022 (21) |
| **STATE-WIDE PREVALENCE** | | | | | | | | |
| **State** | **Year** | **Stunting (%)** | | **Wasting (%)** | | **Underweight (%)** | | **Reference** |
| | | **Urban** | **Rural** | **Urban** | **Rural** | **Urban** | **Rural** | |
| **Ibadan, Oyo** | NA | 30.7 | 40.6 | | | | | Adepoju et al, 2018 (44) |
| **Ibadan, Oyo** | NA | 27.4 | | 1.8 | | | | Yahaya et al 2021 (10) |
| **Lagos** | NA | 12.6 | 64.7 | 8.7 | 9.3 | 9.3 | 26 | Senbanjo et al, 2019 (39) |
| **Benue** | NA | 38.2 | 49.5 | 7.9 | 7.4 | 7.9 | 5.3 | Seer-Uke et al, 2021 (56) |

Supplementary Table 4: Prevalence of Stunting, Wasting, and Underweight According to Gender

| Region | State | Year of data collection | Stunting (%) | | Wasting (%) | | Underweight (%) | | Reference |
|---|---|---|---|---|---|---|---|---|---|
| | | | Male | Female | Male | Female | Male | Female | |
| | | | | **NATIONAL PREVALENCE** | | | | | |
| | | 2013 | 53.1 | 46.9 | 52.3 | 47.7 | 53.5 | 46.5 | Agu et al, 2019 (46) |
| **Nigeria** | | 2017 | 26.9 | 23.1 | 17.5 | 17.5 | 20.5 | 23 | Ogunnaike et al, 2018 (51) |
| | | 2018 | 54.1 | 45.9 | 59.1 | 40.9 | 53.3 | 46.7 | Tesfaw et al, 2021 (19) |
| | | 2002-2020 | 39.4 | 34.2 | - | - | | | Ekholuentale et al, 2022 (45) |

**STATE-WIDE PREVALENCE**

| Region | State | Year | Stunting (%) | | Wasting (%) | | Underweight (%) | | Reference |
|---|---|---|---|---|---|---|---|---|---|
| | | | **Male** | **Female** | **Male** | **Female** | **Male** | **Female** | |
| **North West** | Kaduna & Jigawa | NA | 75.8 | 51.1 | 3 | 8.5 | 6.1 | 10.6 | Kehinde et al, 2022 (22) |
| | Kaduna | NA | 73.2 | 45.7 | 42.5 | 60.9 | 61.5 | 61.4 | Tanimu et al, 2021 (16) |
| | Kaduna | NA | 55.3 | 44.7 | 50.0 | 50.0 | 55.9 | 44.1 | Danimoh et al, 2020(48) |
| **North Central** | FCT, Abuja | 2017 | 44.7 | 37.2 | 29.2 | 29.5 | 46.0 | 37.8 | Idowu et al, 2020 (30) |
| **South East** | Enugu | NA | 19.4 | 21.4 | 12.9 | 10.1 | 18.4 | 17.2 | Amadi et al, 2018 (49) |
| **South West** | Ondo | 2013 | 27.1 | 25.8 | 7.8 | 8.2 | 14.9 | 14.4 | Osunmakinwa et al, 2022 (50) |
| | Ondo | 2018 | 20.2 | 16 | 2.5 | 4.0 | 15.1 | 7.0 | Osunmakinwa et al, 2022 (50) |
| | Ondo | 2019 | 14.4 | 18.7 | 13 | 14.4 | 27.5 | 17.1 | Akande et al, 2019 (53) |
| **South South** | Rivers | NA | | | | | 10.5 | 9.2 | Grace et al, 2019 (40) |
| | Rivers | NA | 13.4 | 14 | 9.2 | 8.3 | 9.2 | 11.9 | Okari et al, 2019 (25) |
| | Edo | 2016-2017 | | | | | 21.8 | 7.9 | Atimati et al 2019 (54) |
| | Edo | 2017 | 34.1 | 30.7 | | | 18.3 | 16.9 | Manyong et al, 2021 (58) |

Supplementary Table 5: Prevalence of Overweight and Obesity in Nigeria

| Region | State | Year of data collection | Overweight % | Obesity % | Reference |
|---|---|---|---|---|---|
| North West | Sokoto | 2017 | 5.6 | 5.3 | Manyong et al, 2021 (58) |
| | Kebbi | 2017 | 17.2 | 16.5 | |
| North Central | Bida, Niger | | 2.6 | 0.9 | Oladele et al, 2022(31) |
| | Benue | NA | 12.1 | 25.9 | Seer-Uke et al. 2021 (56) |
| | Benue | 2017 | 7.8 | 5.8 | Manyong et al, 2021(58) |
| | Abuja FCT | NA | | 0.6 | Idowu et al., 2020 (36) |
| South East | Enugu | 2018 | 9.0 | 2.8 | Ajah et al, 2021(41) |
| | Ebonyi | 2017 | 5.7 | 3.9 | Manyong et al, 2021 (58) |
| | Oyo | | 14.4 | 20.2 | Bamisaye et al., 2018 |
| | Ibadan, Oyo | | 17.2 | 22.3 | Bamisaye et al., 2018 |
| | Ido, Oyo | | 5.0 | 13.9 | Bamisaye et al., 2018 |
| | Lagos | 2013 | 16.6 | | Adeniyi et al, 2018 (57) |
| | Ogun | 2017 | 11.1 | 9 | Okike et al, 2019 (93) |
| | | 2017 | 11.1 | 8.6 | Manyong et al, 2021(58) |
| | Ondo | 2019 | 10.2 | 15.2 | Akande et al, 2019 (53) |
| South South | Rivers | NA | 1.5 | | Okari et al, 2019 (25) |
